# Thymoquinone and 3HQ synergy inhibits CTX-M-15 ESBL

**DOI:** 10.17305/bb.2025.12431

**Published:** 2025-08-11

**Authors:** Karem Ibrahem, Mohammad W Alrabia, Asif Fatani, Sameer E M Alharthi, Hani Zakareya Asfour, Nabil A Alhakamy, Hatoon A Niyazi, Hisham N Altayb, Ahmad M Sait, Philip J R Day, Abdelbagi Alfadil

**Affiliations:** 1 Department of Clinical Microbiology and Immunology, Faculty of Medicine, King Abdulaziz University, Jeddah, Saudi Arabia; 2 Department of Pharmacology, Faculty of Medicine, King Abdulaziz University, Jeddah, Saudi Arabia; 3 Center of Research Excellence for Drug Research and Pharmaceutical Industries, King Abdulaziz University, Jeddah, Saudi Arabia; 4 Department of Pharmaceutics, Faculty of Pharmacy, King Abdulaziz University, Jeddah, Saudi Arabia; 5 Mohamed Saeed Tamer Chair for Pharmaceutical Industries, King Abdulaziz University, Jeddah, Saudi Arabia; 6 Department of Biochemistry, Faculty of Science, King Abdulaziz University, Jeddah, Saudi Arabia; 7 Department of Clinical Microbiology Laboratory, King Abdulaziz University Hospital, Jeddah, Saudi Arabia; 8 Medical Laboratory Science, Faculty of Applied Medical Science, King Abdulaziz University, Jeddah, Saudi Arabia; 9 Regenerative Medicine Unit, King Fahd Medical Research Center, King Abdulaziz University, Jeddah, Saudi Arabia; 10 Division of Evolution and Genomic Sciences, Faculty of Biology, Medicine and Health, The University of Manchester, Manchester, UK; 11 Department of Medicine, University of Cape Town, Cape Town, South Africa

**Keywords:** AMR, ESBL, FICI, 3HQ, thymoquinone

## Abstract

Bacterial infections remain a significant cause of mortality worldwide, further aggravated by the escalating issue of antibiotic resistance. Extended-spectrum beta-lactamases (ESBLs) pose a substantial challenge, capable of hydrolyzing various beta-lactam antibiotics. The slow pace of drug discovery, coupled with the rapid emergence of drug-resistant bacteria, underscores the urgent need for innovative therapeutic solutions. Thymoquinone (TQ), derived from the seeds of *Nigella sativa*, has demonstrated notable antibacterial activity against Gram-negative bacteria, including *Escherichia coli* and *Pseudomonas aeruginosa*. Previous research has established the efficacy of quinoxaline derivatives, such as 3-hydrazinoquinoxaline-2-thiol (3HQ), against methicillin-resistant *Staphylococcus aureus* (MRSA). This study investigates the potential synergy between 3HQ and TQ against various clinical strains of ESBL. The minimum inhibitory concentrations (MICs) of TQ and 3HQ were evaluated against 18 clinical ESBL strains, revealing MIC values ranging from 16 to 128 µg/mL for both compounds. Furthermore, the interaction between TQ and 3HQ was assessed using a checkerboard assay, which demonstrated a 100% synergistic interaction, with a fractional inhibitory concentration index (FICI) of less than 0.5 against the ESBL strains. Docking and molecular dynamics simulations indicated that TQ exhibits a strong binding affinity and interaction profile comparable to that of RPX-7063. In contrast, 3HQ targets a different active site, potentially enhancing thymoquinone’s binding efficiency. Collectively, these compounds may effectively inhibit CTX-M-15, as evidenced by their docking scores and interaction profiles. Further investigations, including *in vivo* studies, are essential to validate these findings. This research suggests a promising strategy for developing more effective treatments for ESBL infections, emphasizing the need for *in vivo* validation.

## Introduction

The persistent threat of bacterial infections significantly contributes to global mortality rates, exacerbated by the escalating issue of antibiotic resistance [1–3]. Extended-spectrum beta-lactamases (ESBLs) present a considerable challenge in modern healthcare settings, primarily due to their association with multidrug-resistant organisms. This relationship necessitates integrated and effective strategies for control and management in healthcare environments [4, 5].

Globally prevalent, ESBLs are particularly common in regions such as Europe and the United States. They are produced by various members of the Enterobacteriaceae family, as well as *Pseudomonas aeruginosa*, and possess the capability to hydrolyze several beta-lactam antibiotics, including aztreonam and third-generation cephalosporins [6–8]. The proliferation of ESBLs has profound implications for treatment strategies, as these enzymes confer resistance to a broad spectrum of antibiotics, particularly within the beta-lactam class. This resistance significantly restricts treatment options and complicates traditional methods for addressing bacterial infections [9, 10].

CTX-M extended-spectrum β-lactamase (ESBL)-producing *Klebsiella pneumoniae* isolates are infrequently reported in the United States. CTX-M-type ESBL enzymes have also been identified in non-*E. coli* Enterobacteriaceae species, including *Klebsiella spp.*, *Proteus mirabilis*, *Enterobacter spp.*, *Salmonella spp.*, *Shigella spp.*, and *Morganella morganii*. Furthermore, the emergence of these enzymes has recently been documented in *Acinetobacter baumannii* and *Pseudomonas aeruginosa* [11, 12].

The slow progress in drug discovery, coupled with the rapid proliferation of drug-resistant bacteria, poses significant concerns for global health. In 2019, antibiotic resistance (AMR) caused 1.27 million deaths worldwide, with the highest rates occurring in sub-Saharan Africa. Six primary pathogens were responsible for the majority of these fatalities, including *Escherichia coli*, *Staphylococcus aureus*, *Klebsiella pneumoniae*, *Streptococcus pneumoniae*, *Acinetobacter baumannii*, and *Pseudomonas aeruginosa*. Notably, methicillin-resistant *S. aureus* alone accounted for over 100,000 deaths, while other significant contributors included drug-resistant *E. coli*, *K. pneumoniae*, and *A. baumannii* [13]. Projections suggest that infections caused by drug-resistant organisms could result in 10 million deaths by 2050. Furthermore, this trend may lead to treatment failures, increased medical costs, extended hospital stays, and heightened socioeconomic burdens [14–16]. Consequently, there is an urgent need for innovative strategies to address these challenges [17].

Combining antibiotics is a well-established, effective, and economically viable approach to combating resistant bacterial infections [18]. Employing a synergistic strategy for drug development, particularly through the repurposing of existing medications instead of creating entirely new compounds, has demonstrated considerable advantages [19, 20]. This method has the potential to generate savings exceeding $1 billion and reduce the time required for FDA approval by 50% [21]. By leveraging existing drugs in novel combinations, researchers can take advantage of their established safety profiles and known pharmacological properties, thereby streamlining the drug development process and accelerating the availability of effective treatments for resistant bacterial infections [22, 23]. This innovative strategy not only addresses the urgent demand for new antimicrobial agents but also provides a practical and efficient pathway to combat the escalating threat of antibiotic resistance [24].

Thymoquinone (TQ), an active constituent extracted from the seeds of *Nigella sativa*, commonly known as black cumin or black seed, is a naturally derived compound with significant biological activity. This bioactive substance is renowned for its medicinal properties and has been utilized for centuries in traditional medicine and culinary practices across various cultures [25]. The natural occurrence of TQ within these seeds underscores its potential as a therapeutic agent, with research investigating its diverse pharmacological effects and potential applications in healthcare and wellness [26].

TQ exhibits notable antibacterial effects against both Gram-negative bacterial strains. Its antimicrobial properties extend to inhibiting biofilm formation, particularly in Gram-negative bacteria such as *Escherichia coli* and *Pseudomonas aeruginosa*. This dual action against bacterial growth and biofilm formation highlights the therapeutic significance of TQ in addressing microbial infections, especially those caused by Gram-negative pathogens known for their resilience and ability to form biofilms, which can exacerbate antibiotic resistance and treatment challenges [27, 28].

A study conducted by Elfadil et al. demonstrated the significant efficacy of quinoxaline derivatives, particularly emphasizing 3-hydrazinoquinoxaline-2-thiol (3HQ), against a diverse range of clinical strains commonly associated with Methicillin-resistant *Staphylococcus aureus* (MRSA) [2, 29]. Furthermore, our research has revealed that 3HQ possesses activity against Gram-negative strains producing ESBL (unpublished data).

Based on prior research, we propose that the combination of quinoxaline derivatives with TQ may enhance their efficacy against various ESBL clinical strains. This innovative approach aims to address the complexities associated with ESBL infections and to open new avenues for treatment. Our study intends to analyze the *in vitro* antimicrobial efficacy of the combination of 3HQ and TQ across diverse ESBL clinical strains. Our objective is to identify potential synergistic effects between these compounds, thereby improving treatment outcomes and presenting novel strategies to combat ESBL infections.

## Materials and methods

### Antibacterial compounds

The compounds tested in this study, specifically 3HQ, were obtained from Fluorochem Ltd. in the United Kingdom. The TQ powder used was sourced from Sigma. For experimental purposes, both compounds were dissolved in a 5% dimethyl sulfoxide (DMSO) solution, also acquired from Sigma. This careful sourcing and preparation process ensured the integrity and reliability of the compounds used in the experimental procedures.

### Bacterial strains, growth media and condition

For this study, bacterial strains were rigorously selected from a pool of 18 ESBL-producing isolates obtained from King Abdulaziz University Hospital in Jeddah, Saudi Arabia. These isolates were preserved in glycerol and stored at –80 ^∘^C to ensure their viability and integrity. Upon retrieval, a thawing process was conducted to optimize the recovery of the bacterial cultures. Subsequently, the isolates were cultured on blood agar plates from HiMedia or MacConkey agar, a reputable supplier based in India. Each isolate underwent identification and susceptibility testing using the Vitek 2 system (bioMerieux, France) with the Gram-negative strain card type AST-N417, adhering strictly to the manufacturer’s guidelines. The cultivation process was carried out overnight at 37^∘^C under aerobic conditions to promote optimal bacterial growth. Notably, ethical approval was not required for this study, as it involved only the analysis of bacterial isolates without any patient-specific data or history. The focus of the study was solely on bacterial specimens, negating the need for ethical clearance.

### Sensitivity test

To evaluate the sensitivity of antimicrobials, we utilized a broth microdilution assay. This procedure began with the preparation of a two-fold serial dilution of the antibiotics under investigation in Mueller–Hinton Broth (MHB), obtained from Sigma-Aldrich, USA. Precise aliquots of 100 µL of the antibiotic solutions were dispensed into each well of 96-well plates sourced from Italy. Each drug’s original stock solution was prepared at a concentration of 10 mg/mL, from which a 128 µg/mL solution was created with MHB. This solution underwent a series of two-fold serial dilutions before being pipetted into the corresponding wells.

To ensure the accuracy of our inoculum suspension, its density was adjusted to 0.5 McFarland units using a Biosan Densitometers DEN-1B turbidity detector. Following this calibration, precise volumes of 5 µL of the prepared inoculum were added to each well containing varying concentrations of antibiotics. The prepared plates were then incubated overnight at 37 ^∘^C. Antibiotic susceptibility testing was conducted in triplicate to enhance the robustness and reliability of the results. Subsequently, mean values were recorded for further analysis and interpretation. This systematic approach ensured the accuracy and reliability of our antibiotic susceptibility testing methodology [2].

### Checkerboard assay

To assess the interactions between antimicrobial agents, the checkerboard broth assay was employed. This method involved preparing a twofold serial dilution of each compound in MHB. Subsequently, 50 µL of each dilution was transferred into a 96-well plate. The inoculum suspension density was precisely adjusted to 0.5 McFarland using a Biosan Densitometer DEN-1B for turbidity detection. Following this, 5 µL of the diluted bacterial suspension was added to each well of the 96-well plate [1]. The checkerboard test was conducted three times, and the average values were recorded for further analysis.

### Assessment of the interactions between the tested antimicrobial agents

A checkerboard assay was employed to evaluate the interactions among antimicrobial drugs. This assay enabled the assessment of all possible combinations of two drugs within a specified concentration range. The interaction between the two drugs was quantitatively analyzed using the fractional inhibitory concentration index (FICI), calculated with the following formula: FICI ═ [(MIC 3HQ in combination)/MIC 3HQ alone] + [(MIC TQ in combination)/MIC TQ alone].

The results of the FICI are interpreted as follows: Values equal to or below 0.5 indicate synergy; values greater than 0.5 but less than or equal to 1 suggest an additive effect; values exceeding 1 but not above 2 indicate indifference; and values above 2 signify antagonism. Practically, synergy, as determined by this calculation, corresponds to a reduction in the MIC of each drug by at least two dilution levels when combined [30, 31].

### Statistical analysis

GraphPad Prism was utilized for statistical analysis. Each experiment included a minimum of three replicates, from which mean and standard deviation (SD) values were calculated. An unpaired *t*-test was conducted to evaluate significant differences between the experimental groups, with a significance threshold set at a *P* value of 0.05 or lower. The *P* values were reported as follows: *P < 0.05, **P < 0.01, and ***P < 0.001. Detailed statistical analysis is provided in the figure legends. Due to the limited sample size, formal normality tests, such as the Shapiro–Wilk test, were not performed; rather, we assumed approximate normality based on the consistent distribution of minimum inhibitory concentration (MIC) values. Unpaired *t*-tests were employed for predefined, hypothesis-driven comparisons. No correction for multiple comparisons was implemented, as the analysis was focused and not exploratory in nature.

### Docking study

In this study, the structure of the CTX-M-15 enzyme bound to the ligand RPX-7063 was obtained from the Protein Data Bank (PDB) under the ID 7TI0 (https://www.rcsb.org/structure/7TI0). This structure, resolved at a resolution of 1.5 Å, includes the ligands TQ and 3HQ, which were accessed via the PubChem database (https://pubchem.ncbi.nlm.nih.gov/). TQ was retrieved with PubChem ID 10281, while 3HQ was obtained with PubChem ID 781224.

Prior to molecular docking, the protein and ligands underwent extensive preparation. All water molecules were removed, and hydrogen atoms were added to both the protein and ligands to accurately represent the protonation states under physiological conditions, a critical step for facilitating hydrogen bond formation during docking [32]. The protein-ligand complexes were subsequently subjected to energy minimization to alleviate steric clashes and ensure that the structures were in their lowest energy conformations. These prepared structures were then utilized in molecular docking studies using the Maestro interface, employing the XP docking protocol to investigate the potential binding modes of TQ and 3HQ with the CTX-M-15 enzyme. This set the stage for further analysis through molecular dynamics (MD) simulations and interaction profiling.

### MD simulations

MD simulations were employed to elucidate the interactions between TQ, 3HQ, and the CTX-M-15 enzyme, as well as to evaluate their potential as inhibitors. These simulations were conducted using the Desmond module within the Maestro platform [33], a comprehensive tool for molecular modeling. The simulations were carried out over a duration of 50 nanoseconds (ns), which is adequate for observing the dynamic behavior of the complexes and assessing their stability over time.

During the simulations, the atomic motions of both the protein and ligands were monitored, facilitating the analysis of critical interactions, including hydrogen bonds, hydrophobic interactions, and water bridges. Root mean square deviation (RMSD) values were computed for the protein and ligands, offering insights into the structural stability of the complexes throughout the simulation period. The resulting data were subsequently utilized to create interaction histograms, which illustrate the frequency and nature of these interactions over the course of the simulation.

## Results

### Assessing the MICs of both 3HQ and TQ

Before proceeding with the checkerboard method, it is essential to determine MICs of both 3HQ and TQ. This preliminary step is crucial as it establishes the baseline effectiveness of each compound against the target pathogen. By accurately determining the MICs, we can ensure optimal concentrations are employed in subsequent combination studies, thereby maximizing the potential synergistic effects between the two compounds. This careful approach lays the foundation for a thorough evaluation of their combined antimicrobial activity, ultimately contributing to a more robust assessment of their efficacy against the target pathogen.

As presented in Table 1, both TQ and 3HQ exhibited MIC values ranging from 16 to 128 µg/mL. Following the acquisition of these MIC values, a well-structured checkerboard test protocol was developed. This approach involved blending varying quantities of TQ and 3HQ, calibrated to investigate potential synergistic interactions between the two compounds.

**Table 1:** Comparative MIC values of 3HQ and TQ (measured in µg/mL) for clinical ESBL species

**Number of strain**	**ESBL producing organism**	**MIC of 3HQ**	**MIC of TQ**
1	*Pseudomonas aeruginosa*	128	128
2	*Klebsiella pneumoniae*	64	128
3	*Klebsiella pneumoniae*	64	128
4	*Escherichia coli*	32	64
5	*Acinetobacter baumannii*	32	128
6	*Escherichia coli*	64	32
7	*Escherichia coli*	32	32
8	*Escherichia coli*	32	32
9	*Escherichia coli*	32	64
10	*Escherichia coli*	32	64
11	*Escherichia coli*	32	16
12	*Escherichia coli*	16	32
13	*Escherichia coli*	32	64
14	*Escherichia coli*	32	32
15	*Escherichia coli*	32	16
16	*Escherichia coli*	64	32
17	*Escherichia coli*	32	32
18	*Escherichia coli*	16	16

Abbreviations: MICs: Minimum inhibitory concentrations; ESBLs: Extended-spectrum β-lactamases; 3HQ: 3-Hydrazinoquinoxaline-2-thiol; TQ: Thymoquinone.

### 3HQ and TQ exhibit synergistic effects against diverse ESBL clinical strains

**Figure 1.**
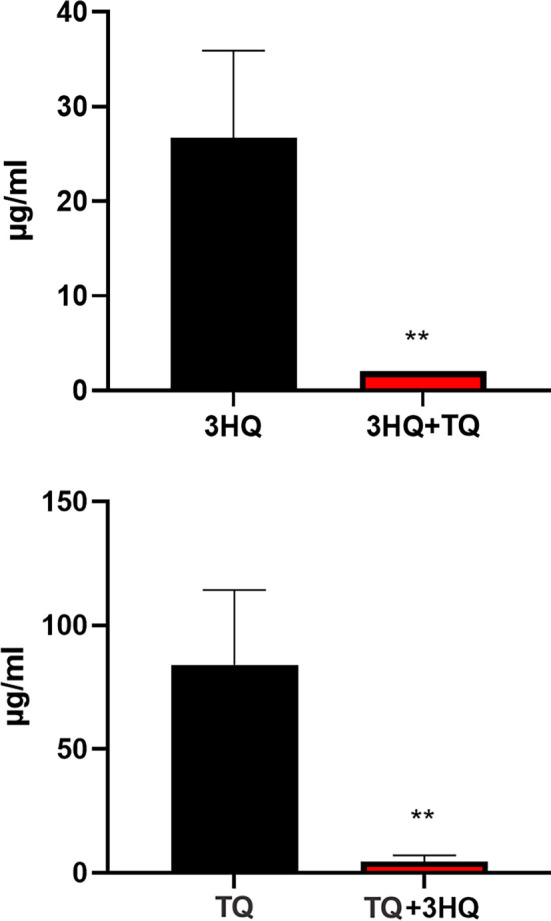
**Minimum inhibitory concentrations (MICs) of 3-hydrazinoquinoxaline-2-thiol (3HQ), thymoquinone (TQ) and their combination against the tested isolate, *Escherichia coli* strain 4.** Statistical comparisons were performed using unpaired *t*-tests. Significant differences are indicated by asterisks on the bars: *P < 0.05, **P < 0.01, ***P < 0.001. The MICs for 3HQ combined with TQ show a *P* value of 0.0098 when compared to the MICs of 3HQ alone. Similarly, the MICs for TQ combined with 3HQ have a *P* value of 0.002 when compared to the MICs of TQ alone. Data are presented as mean ± standard deviation (SD) from triplicate experiments. A *P* value of less than 0.05 is considered statistically significant.

To evaluate the potential synergistic effects of the combination of 3HQ and TQ against various ESBL clinical strains, a comprehensive checkerboard assay was performed. In this study, the MICs of TQ and 3HQ alone ranged from 16 to 128 µg/mL for inhibiting ESBL growth. However, the most compelling results emerged when these two compounds were combined. The MICs of 3HQ decreased by a factor of 4–16 when paired with TQ against ESBL clinical isolates. Similarly, the MICs of TQ exhibited notable reductions, decreasing by 4–8 times compared to its standalone values across all tested ESBL strains (as illustrated in Figure 1). For instance, in the *Escherichia coli* strain 4, the MIC of 3HQ significantly decreased from 32 µg/mL to 4 µg/mL when combined with TQ, while the MIC of TQ was reduced from 64 µg/mL to 8 µg/mL in the presence of 3HQ, indicating a pronounced synergistic effect.

These findings strongly suggest that 3HQ enhances the efficacy of TQ against ESBL strains, as summarized in Table 2. This phenomenon underscores the potential for synergistic interactions between these compounds in combating ESBL infections, particularly against ESBL strains (Table 2).

**Table 2:** Synergy assessment of 3HQ and TQ vs ESBL strains determined by FICI analysis

**Number of strain**	**ESBL producing organism**	**FIC of 3HQ**	**FIC of TQ**	**FICI**
1	*Pseudomonas aeruginosa*	0.146	0.169	0.315
2	*Klebsiella pneumoniae*	0.167	0.198	0.365
3	*Klebsiella pneumoniae*	0.167	0.169	0.365
4	*Escherichia coli*	0.125	0.125	0.25
5	*Acinetobacter baumannii*	0.208	0.125	0.333
6	*Escherichia coli*	0.125	0.188	0.313
7	*Escherichia coli*	0.188	0.156	0.344
8	*Escherichia coli*	0.2	0.291	0.491
9	*Escherichia coli*	0.084	0.094	0.178
10	*Escherichia coli*	0.149	0.125	0.274
11	*Escherichia coli*	0.146	0.167	0.313
12	*Escherichia coli*	0.167	0.177	0.344
13	*Escherichia coli*	0.146	0.188	0.334
14	*Escherichia coli*	0.104	0.208	0.312
15	*Escherichia coli*	0.167	0.094	0.261
16	*Escherichia coli*	0.208	0.146	0.354
17	*Escherichia coli*	0.208	0.208	0.416
18	*Escherichia coli*	0.167	0.208	0.375

Synergy is considered present when the FICI is less than 0.5. Abbreviations: FIC: Fractional inhibitory concentration; FICI: Fractional inhibitory concentration index; ESBLs: Extended-spectrum β-lactamases; 3HQ: 3-Hydrazinoquinoxaline-2-thiol; TQ: Thymoquinone.

### Molecular docking

**Figure 2.**
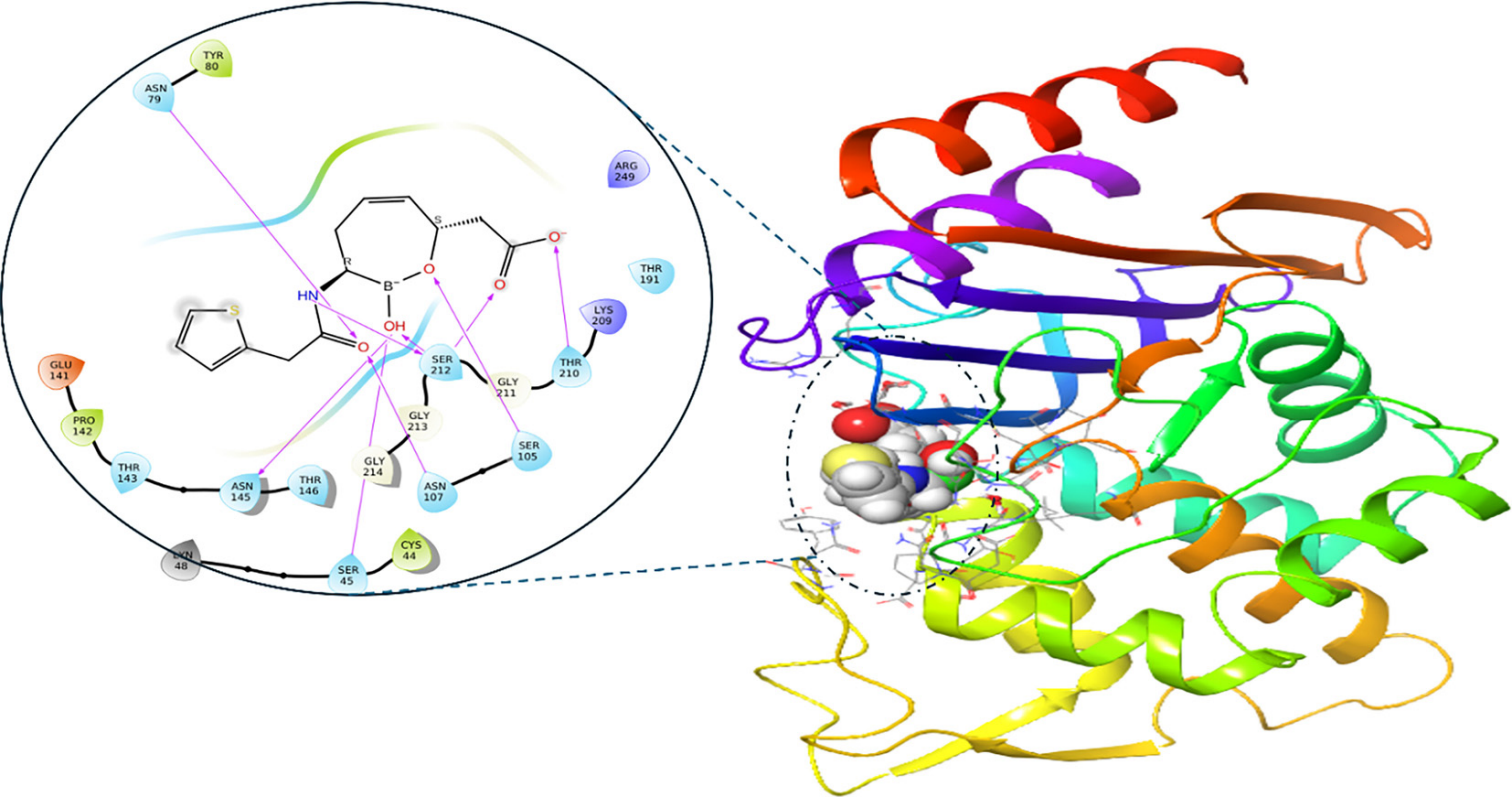
**3D and enlarged 2D representation of the interaction of CTX-M-15 protein and co-crystalized inhibitor (RPX-7063).** In the 3D representation (right), the protein is shown as a rainbow-colored cartoon ribbon, where each color represents a different region of the protein’s secondary structure. The ligand RPX-7063 is displayed in space-filling (ball) representation with carbon atoms in gray, oxygen in red, nitrogen in blue, and sulfur in yellow. In the 2D interaction diagram (left), hydrogen bonds are indicated by purple arrows, pointing from donor to acceptor. Hydrophobic interactions are shown as curved light lines, while amino acid residues are labeled and colored by property.

The docking study investigates the potential synergistic effects of TQ and 3HQ in inhibiting the CTX-M-15 protein. RPX-7063 was utilized as a control to validate our docking protocol. The 2D interaction analysis of the control, depicted in Figure 2, reveals a robust network of interactions that stabilize its binding to the CTX-M-15 protein. RPX-7063 forms multiple hydrogen bonds with critical amino acids, including Ser212, Gly211, Thr210, and Ser105. Additionally, it engages in hydrophobic interactions with residues such as Tyr80 and Asn79, further enhancing its binding affinity. This comprehensive interaction profile underscores the effectiveness of RPX-7063 in binding to the CTX-M-15 protein, establishing it as a benchmark for evaluating other potential inhibitors.

**Figure 3.**
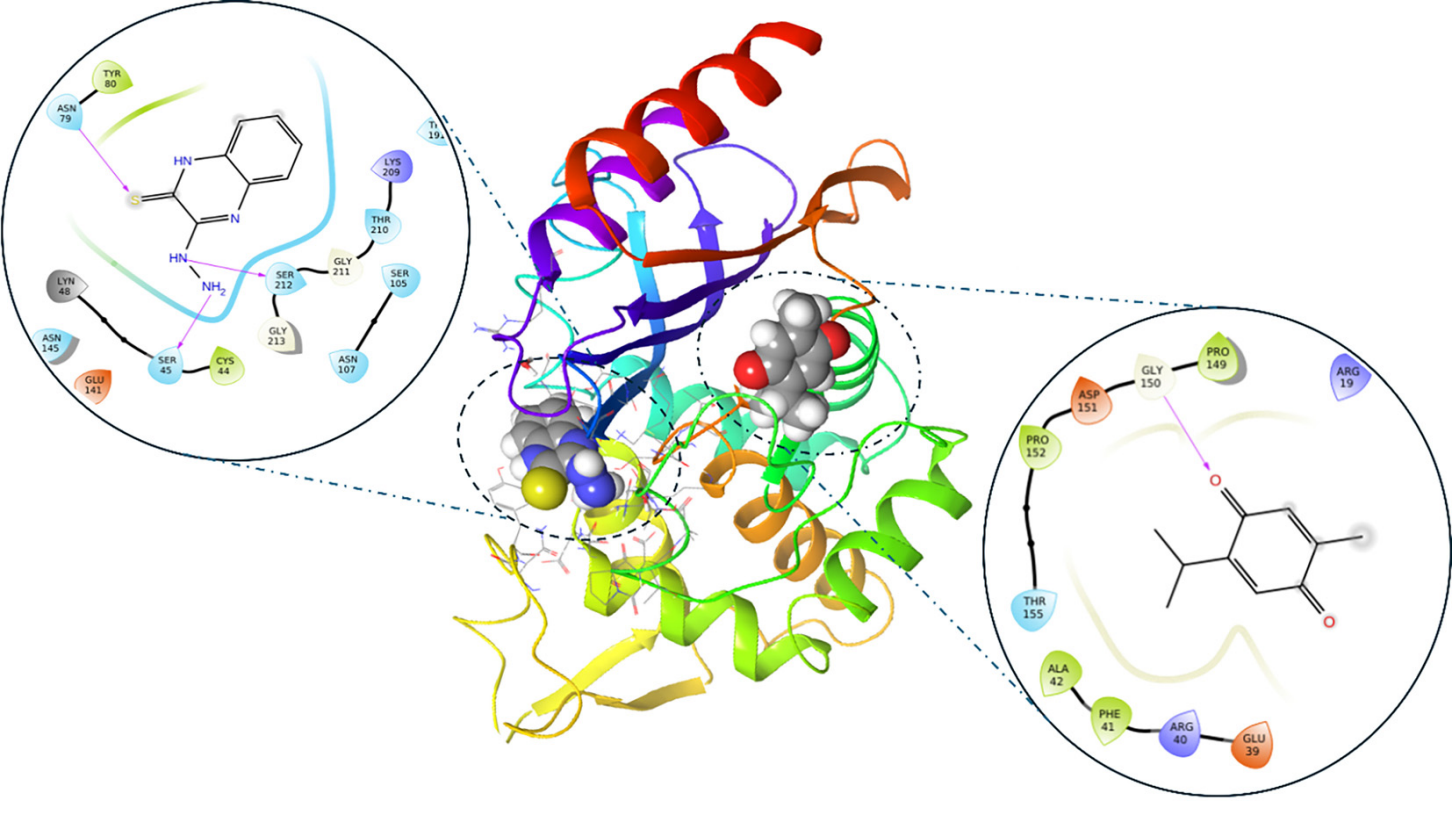
**3D and enlarged 2D representation of the interaction of CTX-M-15.** Purple lines indicate hydrogen bonds, the ligand in 3D representation is shown in balls, while protein is shown in cartoon colored representation. On the upper left enlarged shot showed 3-Hydrazinoquinoxaline-2-thiol interacting with protein main active site, while the lower enlarged shot showed thymoquinone interacting with other binding grove on the protein.

The binding interaction profile of compound 3HQ exhibits similarities to that of RPX-7063. The 2D interaction graphic illustrates that, similar to the control ligand, 3HQ forms hydrogen bonds with critical residues, including Ser212 and Ser45. Additionally, it establishes hydrophobic interactions with residues such as Tyr80 and Asn79, which are essential for maintaining binding stability (Figure 3). Both 3HQ and RPX-7063 display comparable interaction patterns, suggesting that 3HQ may serve as a potent inhibitor of the CTX-M-15 protein, whether administered alone or in combination with other agents. The ability of 3HQ to replicate the control’s interactions indicates its potential for effective binding and inhibition of the protein’s activity. This hypothesis is further supported by a docking score of −3.56 (Table 3), which indicates a favorable binding affinity and corroborates the visualized interaction profile.

In contrast, TQ exhibits a distinct interaction profile, demonstrating fewer overall interactions compared to both RPX-7063 and 3HQ. However, the extent of hydrogen bonding and hydrophobic interactions in TQ is less pronounced. Notably, TQ binds to a unique active site on the CTX-M-15 protein, which may facilitate a potential synergistic effect when combined with 3HQ. This binding at an alternative site could alter the stability or conformation of the protein, thereby enhancing the binding affinity of 3HQ at the primary active site. When used in conjunction with 3HQ, TQ yields a docking score of −3.8, which, while slightly higher than that of 3HQ, indicates a significant binding affinity that may contribute to the overall suppression of CTX-M-15.

**Table 3:** Docking scores for compound–protein interactions

**Compound**	**PubChem ID**	**Docking score**
Thymoquinone	10281	−3.80
3-Hydrazinoquinoxaline-2-thiol	781248	−3.56
RPX-7063	–	−6.9

### MD simulation study

**Figure 4.**
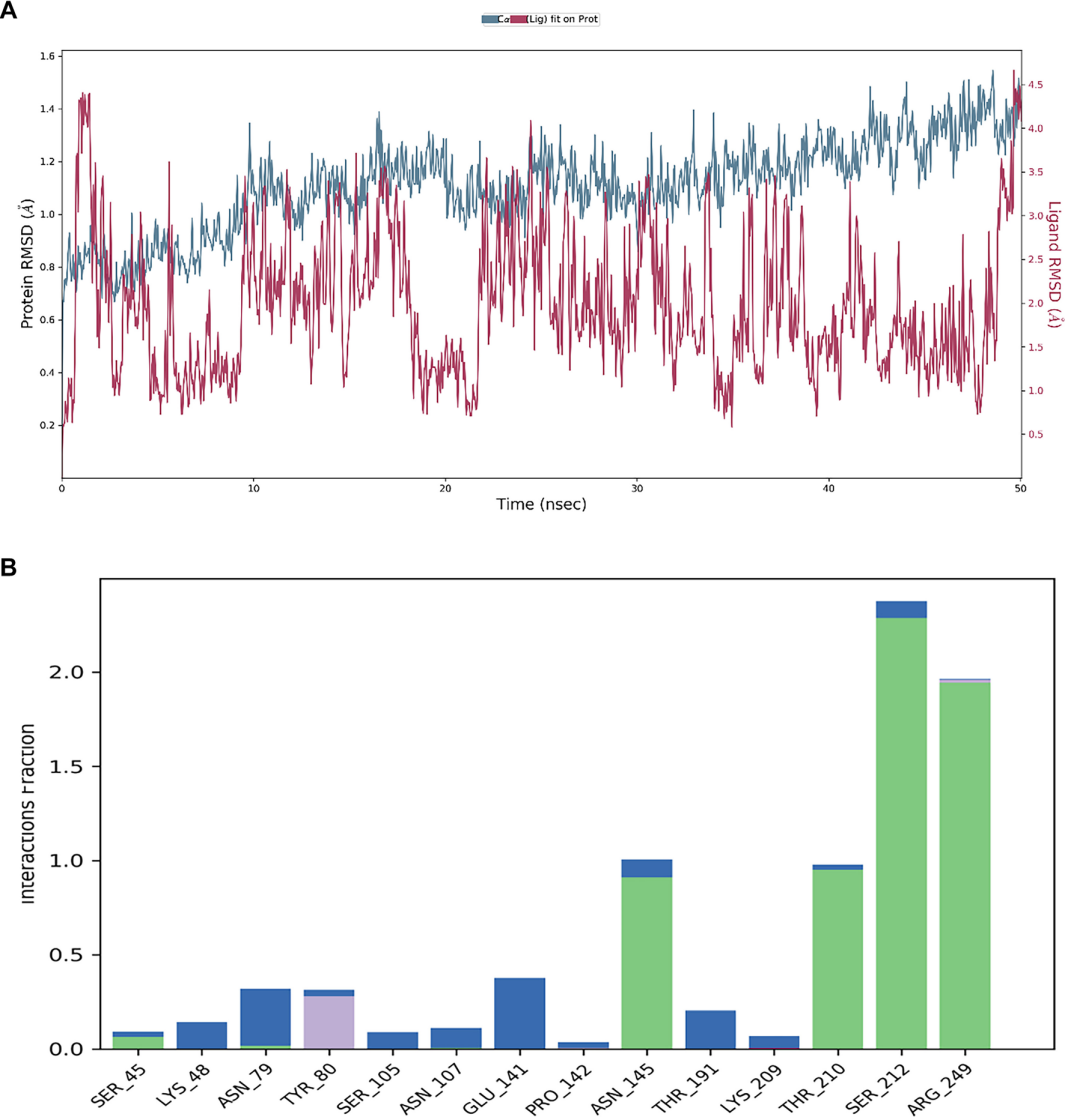
(A) The RMSD for the protein (blue line) for the control compound RPX-7063 (red) and (B) A varied interaction profile is also shown by the notable contributions from hydrogen bonds (green), hydrophobic contacts (purple bars) and water bridges (blue bars). Abbreviation: RMSD: Root mean square deviation.

**Figure 5.**
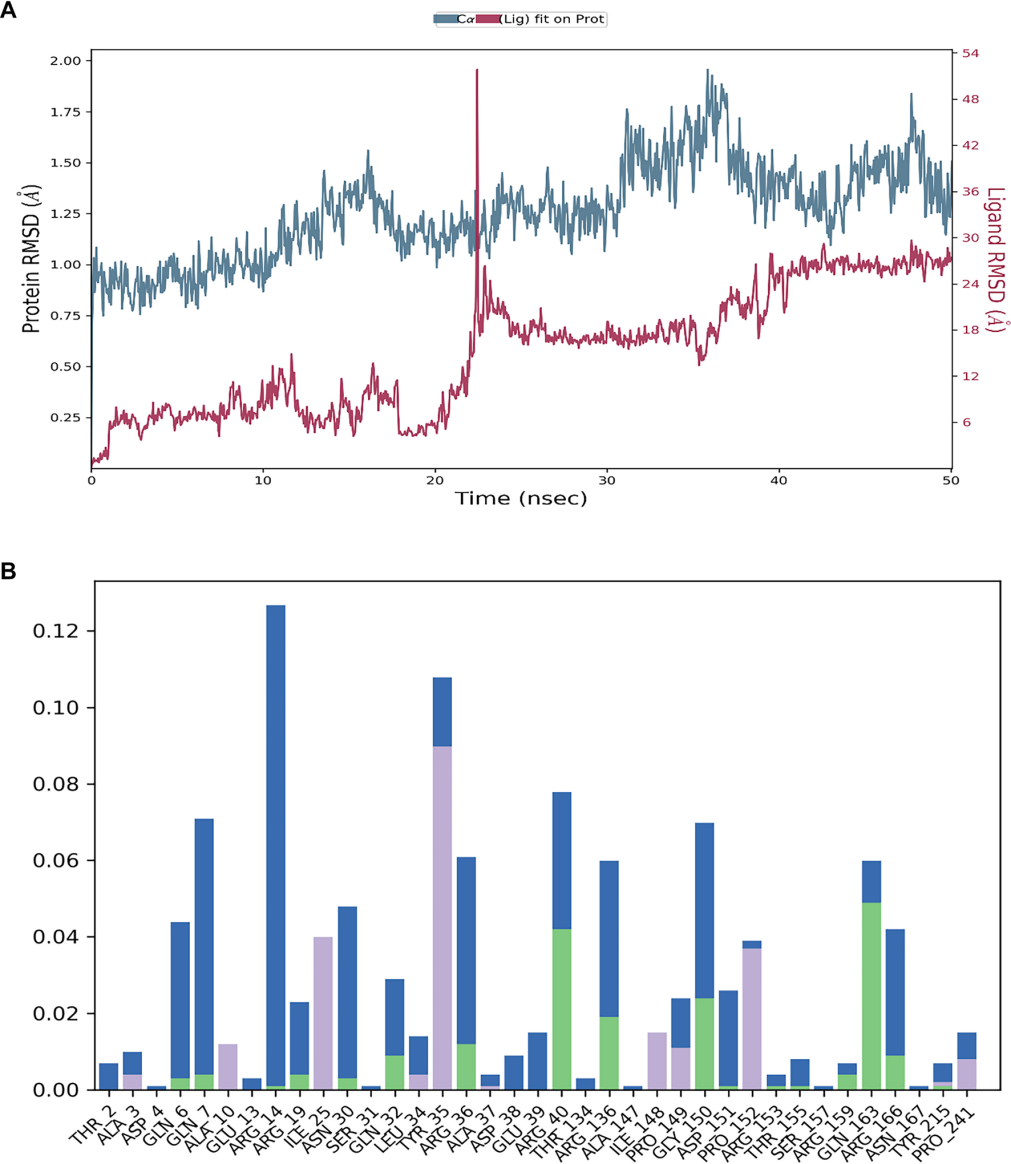
(A) The RMSD for the protein (blue line) for the compound thymoquinone (red) and (B) A varied residual interaction profile is also shown by the notable contributions from hydrogen bonds (green), hydrophobic contacts (purple bars) and water bridges (blue bars). Abbreviation: RMSD: Root mean square deviation.

**Figure 6.**
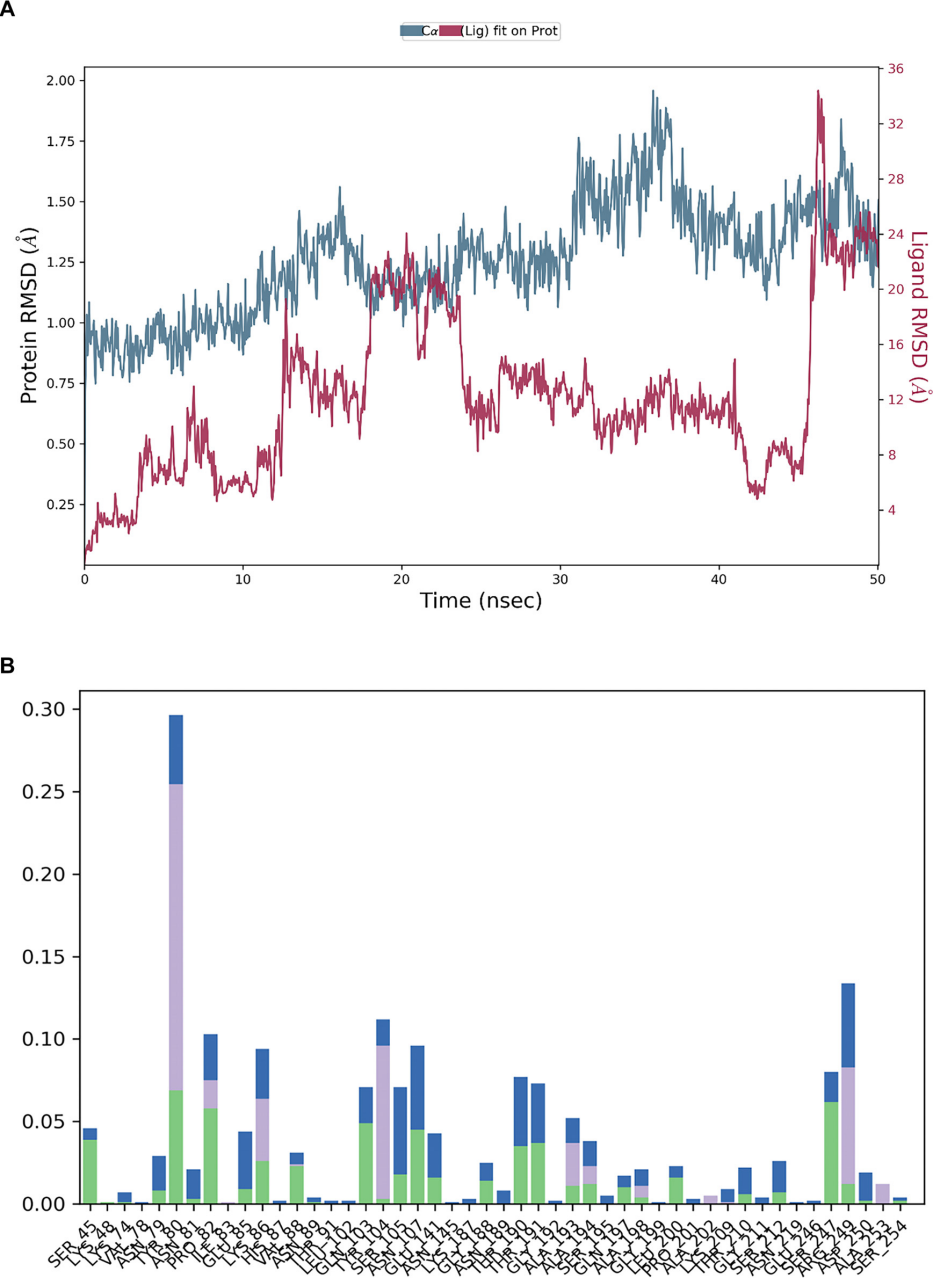
(A) The RMSD for the protein (blue line) for compound 3-Hydrazinoquinoxaline-2-thiol (red) and (B) A varied interaction residual profile is also shown by the notable contributions from hydrogen bonds (green), hydrophobic contacts (purple bars) and water bridges (blue bars). Abbreviation: RMSD: Root mean square deviation.

The stability of the protein-ligand complex over time is illustrated by the RMSD graphs (Figures 4A–6A). The RMSD of the protein (blue line) in the presence of the control compound RPX-7063 (red line) remains relatively stable, suggesting that the protein structure is well-maintained throughout the simulation. Although the ligand’s RMSD (red line) exhibits normal fluctuations, it ultimately stabilizes over time, indicating a consistent interaction with the protein (Figure 4A). A similar trend is observed in the RMSD profile of TQ. The protein demonstrates stability, with the RMSD remaining below 2 Å, which suggests that the protein-ligand complex is well-stabilized [34]. Although the ligand’s RMSD fluctuates initially, it eventually stabilizes, indicating that TQ achieves a steady binding conformation during the simulation (Figure 5A).

In contrast, 3HQ exhibits greater fluctuations in both protein and ligand RMSD, particularly in the ligand, suggesting that the binding may be less stable or that the ligand experiences significant conformational changes throughout the simulation. This variability may arise from its interaction with a different active site compared to TQ and the control compound, potentially influencing the overall stability of the complex (Figure 6B).

The histograms presented in Figures 4B and 5B illustrate the types and frequencies of interactions occurring during the 50 ns simulation. The histogram for the control compound RPX-7063 reveals a high frequency of hydrogen bonding (green bars), which is critical for the stability of the protein-ligand complex. TQ’s histogram displays a similar pattern to that of the control, indicating a substantial number of hydrogen bonds and water bridges. This observation suggests that TQ, akin to the control, establishes a stable and consistent interaction network within the CTX-M-15 binding site. Furthermore, the presence of hydrophobic interactions further enhances the stability of the complex.

Conversely, the histogram for 3HQ indicates a lower frequency of hydrogen bonds and water bridges than both the control compound (RPX-7063) and TQ. This reduced interaction frequency, particularly regarding hydrogen bonds, correlates with the observed higher RMSD fluctuations, suggesting a less stable interaction. Nonetheless, the presence of hydrophobic interactions, albeit infrequent, implies that specific regions may still exhibit stable binding, particularly at various targeted active sites.

Analysis of residue-ligand interactions reveals that both 3HQ and the control compound occupy the canonical active site of the CTX-M-15 enzyme. The control ligand interacts with critical active site residues, including Asn145, Thr210, Ser212, and Arg249, while 3HQ similarly engages residues such as Tyr80, Pro82, Lys86, Tyr104, Asn107, and Arg249, indicating that it binds within the same functional pocket involved in enzymatic catalysis. In contrast, TQ binds to a distinct site that includes residues Arg14, Tyr35, Arg40, Arg136, Gly150, and Gln163, which is spatially separated from the catalytic center. This suggests that TQ may target an allosteric pocket, potentially modulating enzyme activity indirectly. The binding of TQ at this secondary site may induce conformational changes that enhance the accessibility or binding efficiency of 3HQ. MD simulations demonstrate increased RMSD fluctuations in the protein backbone when TQ is bound. This could allosterically modulate the geometry of the active site, thereby enhancing the binding affinity and stability of 3HQ, as evidenced by the lower RMSD and more consistent hydrogen bond formation in combined binding scenarios. This hypothesis is consistent with the observed synergistic reduction in MICs and FICI values.

## Discussion

This study represents the first documented evidence of a synergistic interaction between TQ and 3HQ derivatives against various ESBL clinical strains. This finding enhances our understanding of ESBL therapeutics and suggests a promising strategy for combating antibiotic resistance. The concomitant administration of TQ and 3HQ led to a significant reduction in the MIC of TQ, with decreases observed of up to eightfold. Similarly, the co-administration of TQ with 3HQ resulted in a reduction of the MIC of the 3HQ derivatives by as much as sixteenfold. This robust synergistic interaction was consistently evidenced across experiments involving diverse clinical ESBL strains. These results indicate that the combined therapeutic regimen of TQ and 3HQ elicits a more potent antimicrobial response against ESBL strains than the individual efficacy of each compound alone. Previous research has indicated that incorporating a second antibiotic into treatment regimens can mitigate the limitations of the primary antibiotic [24, 35]. This aligns with our findings, which demonstrate that the drug combination significantly reduced the MICs by up to sixteenfold.

The MIC of TQ alone was 64 µg/mL, whereas that of 3HQ was 32 µg/mL. However, when TQ and 3HQ were combined, only 8 µg/mL of TQ and 2 µg/mL of 3HQ were necessary to achieve the same inhibitory effect against *Escherichia coli*. This indicates that the combination therapy results in lower MICs, suggesting that effective therapeutic outcomes can be attained with reduced drug dosages, potentially minimizing the risk of adverse effects. Further investigations are required to confirm and elaborate on this promising finding. Given the potential toxicity associated with high concentrations of 3HQ, TQ, and other pharmaceuticals, an emerging strategy involves utilizing lower doses of each drug synergistically to address this concern [31]. The efficacy of TQ has also been demonstrated against *Proteus vulgaris* [36, 37]. We propose that the combination of antimicrobial therapies could expand the spectrum of coverage; however, additional studies are necessary to validate this hypothesis.

Our research has revealed a significant synergy between the combination of 3HQ and TQ, particularly regarding their effects on a diverse range of clinical ESBL strains. This synergistic effect is multifaceted, arising from several mechanisms that concurrently inhibit bacterial growth. TQ emerges as a key contributor in this relationship, exerting its antimicrobial effects through a series of actions that primarily target the structure and function of bacterial cells. A notable mechanism involves the induction of irreversible damage to bacterial morphology, beginning with the compromise of cell membrane integrity. This disruption leads to the leakage of essential cellular components, particularly proteins vital for bacterial survival. Additionally, TQ penetrates the intracellular domain, disrupting critical proteins necessary for various cellular processes [38–40].

In contrast, our investigation has revealed the critical role of 3HQ in inhibiting DNA synthesis within bacterial cells. This finding elucidates a fundamental mechanism through which 3HQ exerts its antimicrobial effects. By targeting DNA synthesis, 3HQ disrupts the essential processes required for bacterial replication and proliferation. The inhibition of DNA synthesis represents a strategic approach to combating bacterial infections, as it directly obstructs the ability of bacteria to reproduce and spread. Through its effects on DNA synthesis, 3HQ effectively interferes with the replication of genetic material within bacterial cells, ultimately leading to their demise [41–43].

This underscores the multifaceted nature of the combination of 3HQ and TQ in combating bacterial pathogens. While TQ disrupts bacterial morphology and cellular functions, 3HQ acts at the genetic level to inhibit DNA synthesis. Together, these complementary mechanisms synergistically enhance the antimicrobial efficacy of the combination, providing promising avenues for the development of novel therapeutic interventions against bacterial infections.

Another potential explanation for the increased efficacy observed with the combination of TQ and 3HQ against various ESBL clinical strains is the enhanced generation of reactive oxygen species (ROS) [44]. ROS are highly reactive molecules that disrupt essential cellular processes within bacterial cells. A significant consequence of ROS is their interference with cellular electron transport, which triggers a cascade of events that results in sustained oxidative stress and ultimately leads to cell death [45, 46].

The ability of TQ to induce the formation of ROS is well-documented in numerous studies. This property is crucial for its antimicrobial activity, as ROS serve as potent agents against bacterial pathogens. By triggering ROS production, TQ effectively launches a series of oxidative assaults on bacterial cells, overwhelming their defense mechanisms and rendering them susceptible to destruction [27, 47]. When combined with 3HQ, TQ’s capacity to induce ROS formation may be further enhanced, resulting in a more pronounced antimicrobial effect against ESBL strains. This synergistic interaction between TQ and 3HQ underscores the complexity of their mechanisms and highlights the diverse strategies through which they target bacterial pathogens.

The enhanced generation of ROS represents a significant aspect of the multifaceted approach employed by TQ and 3HQ in combating ESBL infections. By exploiting the oxidative vulnerabilities of bacterial cells, this combination holds considerable promise for the development of novel therapeutic strategies against drug-resistant pathogens. Furthermore, this study investigates the potential synergistic effects of TQ and 3HQ on inhibiting the CTX-M-15 protein, using RPX-7063 as a control. Docking and MD simulations revealed that TQ exhibits a strong binding affinity and interaction profile comparable to RPX-7063, forming hydrogen bonds and hydrophobic interactions with critical residues. In contrast, 3HQ binds to a distinct active site, potentially enhancing TQ’s binding efficacy. This combination may effectively inhibit CTX-M-15, as indicated by docking scores and interaction profiles. The findings of this study suggest that the concurrent use of TQ and 3HQ could represent a promising strategy to combat CTX-M-15-mediated antibiotic resistance.

Further testing is essential to comprehensively characterize the efficacy of the combination of TQ and 3HQ against ESBL strains. A critical component of this evaluation is the Time Kill assay, which is necessary for thoroughly examining the bactericidal effects of the TQ and 3HQ combination over a specified duration. This assay will yield valuable insights into the potential of this combination as a long-term treatment strategy [1]. Additionally, a resistance assay is vital to assess the likelihood of bacteria developing resistance to the combination, ensuring the sustained effectiveness of the treatment by identifying potential resistance development [31]. Furthermore, a proteomic analysis is crucial for achieving a comprehensive understanding of the genetic responses when bacteria are exposed to the TQ and 3HQ combination. This analysis will identify the genes that are upregulated or downregulated, thereby elucidating the underlying mechanisms responsible for the observed synergy.

TQ has demonstrated significant antibiofilm activity against *P. aeruginosa*, prompting further investigation into the combined efficacy of TQ and 3HQ against ESBL biofilm formation [27]. This study primarily focuses on *E. coli* strains, with only single isolates of *A. baumannii*, *K. pneumoniae*, and *P. aeruginosa*. Therefore, it is crucial to include a wider variety of strains in future research. Such an expansion will allow for a more thorough assessment of the efficacy of this combination against resistant and challenging organisms. By broadening the spectrum of tested organisms, we can achieve a more comprehensive evaluation of the therapeutic potential of the TQ and 3HQ combination, ultimately leading to more robust treatment strategies for combating resistant bacterial infections. Future investigations will also examine the *in vivo* efficacy and pharmacodynamic properties of this combination to enhance our understanding of its therapeutic applicability.

### Limitation

One limitation of our study is the disproportionate representation of bacterial strains, as we included a substantial number of *E. coli* isolates, while only a single isolate each of *K. pneumoniae*, *P. aeruginosa*, and *A. baumannii*. This imbalance resulted from our reliance on the hospital for the provision of isolates, reflecting the strains that infected patients at the time of our request. Additionally, this study was confined to *in vitro* assessments of the antibacterial activity of TQ and 3HQ. Consequently, further preclinical studies are essential to evaluate the safety and therapeutic potential of this combination.

## Conclusion

Our study offers the first evidence of synergy between 3HQ and TQ against a variety of ESBL strains. Although these findings indicate promising clinical applications, additional tests and a comprehensive proteomic analysis are necessary to further characterize and understand the full potential of this combination. Moreover, *in vivo* studies are essential to assess their toxicity, pharmacokinetics, and overall suitability as potential therapeutic agents.
